# Modelling the potential financial impacts of expanding access to immune checkpoint inhibitors as monotherapy for treating advanced non-small cell lung cancer

**DOI:** 10.1016/j.eclinm.2025.103261

**Published:** 2025-05-24

**Authors:** Kiu Tay-Teo, Dario Trapani, Manju Sengar, Zeba Aziz, Filip Meheus, André Ilbawi, Elisabeth G.E. de Vries, Lorenzo Moja

**Affiliations:** a World Health Organization, Geneva, Switzerland; b European Institute of Oncology, IRCCS, Milan, Italy; c Department of Oncology and Haemato-Oncology, University of Milano, Milan, Italy; d Department of Medical Oncology, Tata Memorial Hospital, Mumbai, India; e Hameed Latif Hospital, Lahore, Pakistan; f Department of Medical Oncology, University Medical Center Groningen, University of Groningen, Groningen, the Netherlands

**Keywords:** Non-small cell lung cancer, Immune checkpoint inhibitor, Access to medicines, Economics, Finances

## Abstract

**Background:**

Access to immune checkpoint inhibitors remains limited due to cost-effectiveness and affordability concerns. This study evaluates the financial impacts of expanding global access to PD1/PD-L1 inhibitors as first-line monotherapy for patients aged 40–74 years with advanced unresectable non-small cell lung cancer (NSCLC), with wildtype EGFR and ≥50% of tumour cells with PD-L1 expression.

**Methods:**

The potential usage and associated costs were assessed from 2024 to 2040 through repeated cross-sectional assessments. The base case assumed treatment rates in countries with access to PD1/PD-L1 inhibitors in 2023, while expanded-access scenarios projected coverage increases to 30% in low-income, 50% in lower-middle-income, 80% in upper-middle-income (UMICs), and 95% in high-income countries over 10 years.

**Findings:**

The model estimated that 200,000–250,000 individuals are treatment-eligible, with only about one-fifth receiving PD1/PD-L1 inhibitors in the base case. Expanding access would increase global treatment coverage to 75% by 2040, particularly in middle-income countries. The largest increases would be in UMICs (+100,700) and the Western Pacific region (+82,400). At an estimated per-patient lifetime cost of US$37,600–US$75,100, total costs could reach US$14,087 million with fixed dosing, or US$9080 million with weight-based dosing. PD-L1 testing costs would add <1% to the total.

**Interpretation:**

Expanding access to PD1/PD-L1 inhibitors for advanced NSCLC over 10 years demands significant funding, making equitable access in lower-income countries doubtful without a significant price reduction. Policymakers should negotiate lower prices to ensure cost-effectiveness and affordability, improve spending efficiency by optimised dosing and treatment duration, and enhance health system capacity, including ensuring appropriate use and introducing biosimilars.

**Funding:**

This publication was made available as open access through 10.13039/100004423WHO funding provided by two projects: the Universal Health Coverage Partnership (Award 74812, the 10.13039/501100000780European Union, the Grand Duchy of Luxembourg, 10.13039/100009099Irish Aid, the Government of Japan, the 10.13039/501100003388French Ministry for Europe and Foreign Affairs, the 10.13039/501100020171United Kingdom’s Foreign, Commonwealth & Development Office, the Government of Belgium, the 10.13039/501100000023Government of Canada, and the Government of Germany) and the Increasing Global Equitable Access to Health Products & Health Technologies project (Award 72913, the Government of Belgium).

**Research in context**

**Evidence before this study**

We searched PubMed database on February 5, 2025, using the term ((PD-1 OR PD-L1) OR (pembrolizumab OR Keytruda) OR (atezolizumab OR Tecentriq) OR (cemiplimab OR Libtayo)) AND (monotherap∗) AND (advanced OR metastatic) AND (first line OR previously untreated) AND (non-small cell lung cancer OR NSCLC) AND ((costs and cost analysis) OR (“budget impact analysis”)), without language restrictions. We identified 16 articles evaluating the cost-effectiveness of immune checkpoint inhibitors for advanced NSCLC from seven countries, all of which were high-income countries except for China. All studies reported incremental cost-effectiveness ratios exceeding the cost-effectiveness threshold of at least the per capita Gross Domestic Product, indicating that immune checkpoint inhibitors were not deemed value for money at the market price despite their superior efficacy compared to chemotherapy. Only per-patient costs were described, and no budget impact analysis was presented.

**Added value of this study**

This modelling study assessed the potential financial impacts of expanding global access to PD1/PD-L1 inhibitors as first-line monotherapy for NSCLC, focusing on patients most likely to benefit: individuals aged 40–74 years with advanced unresectable NSCLC, wildtype *EGFR*, and ≥50% tumour cells expressing PD-L1. The study found that expanding access to reach target coverage rates of 30%–95% over the next 10 years could increase global treatment coverage for eligible patients from an estimated 22% in 2024 (primarily concentrated in high-income countries) to as high as 75%, with a particularly notable increase in middle-income countries. Such expansion would incur an annual cost of up to US$14,087 million with fixed dosing or US$9080 million with weight-based dosing.

**Implications of all the available evidence**

Expanding global access to PD1/PD-L1 inhibitors for advanced NSCLC requires substantial financial commitments, casting doubts about the feasibility of achieving equitable access in lower-income countries unless prices are significantly reduced. Lower prices are crucial not only for affordability but also for cost-effectiveness. To address these challenges, policymakers should negotiate substantially lower prices, optimise dosing and treatment duration to improve spending efficiency, and strengthen health system capacity to ensure appropriate use and early introduction of quality-assured biosimilars.

## Introduction

The introduction of immune checkpoint inhibitors since 2011 has transformed the cancer treatment landscape. By 2024, regulatory agencies have approved over 15 immune checkpoint inhibitors directed to CTLA4, PD1, PD-L1, and LAG3, approved for use as single agents (except LAG3 inhibitors) or in combination with other treatments to manage a wide range of solid tumours and haematological malignancies. Approvals have predominantly focused on advanced cancers but there is a growing trend towards using these medicines for earlier-stage disease.^1^ With over 5000 clinical trials underway,^2^ the number of approved immunotherapies, treatment combinations, and indications is expected to expand rapidly in the coming years. This might offer more therapeutic options, but will introduce greater complexity to clinical service delivery. Commercially, the global market for immune checkpoint inhibitors showed significant growth, with PD1/PD-L1 inhibitors generating US$52 billion in sales in 2023, and projected to exceed US$90 billion by 2028.^1^

Despite the scientific and commercial successes of immune checkpoint inhibitors, patient access in many countries remains limited or absent. This disparity is reflected in the concentration of usage in North America, Europe, and Japan.^1^ Specifically, seven countries – the United States of America, France, Germany, Italy, Spain, the United Kingdom, and Japan – account for about 80% of the global sales^3^ while representing less than a third of the newly diagnosed cases of cancers globally.^4^ Limited access to these medicines, especially in low- and middle-income countries, underscores a multitude of systemic barriers. One significant access barrier is the pricing of immune checkpoint inhibitors, which far exceeds the financial capacity of many public healthcare systems and individuals, leading to catastrophic out-of-pocket expenses, treatment interruptions, or even abandonment of care. In high-income countries with universal insurance coverage for medicines, the long-term sustainability of funding for PD1/PD-L1 inhibitors has raised concerns, as these medicines already account for up to a quarter of total cancer medicine expenditures.^1^^,^^3^ In response, various strategies have been proposed and measures implemented to enhance efficiency. These include dose optimization according to patient weight (weight-based dosing), rounding down doses to the closest vial size and strength (‘dose banding’), shortening treatment duration, vial-sharing to reduce waste, considering therapeutic interchangeability (‘class effect’), and preferential pricing and prescribing.^5^, ^6^, ^7^, ^8^, ^9^, ^10^, ^11^, ^12^, ^13^ Studies suggested that these measures may reduce costs by 43%–50% for pembrolizumab.^5^^,^^6^^,^^10^ However, additional strategies should be implemented to mitigate the intensifying financial burden on national health systems and individuals, and the widening inequitable access gap both between and within countries, as high-cost immune checkpoint inhibitors proliferate and treatment complexity rises in coming years.

The World Health Organization (WHO) Model List of Essential Medicines (EML) serves to guide countries in selecting medicines with established efficacy and safety that are relevant to public health. Including medicines on the EML signals clinical and resource allocation priorities, which might accelerate their adoption in countries, provided financial resources are available, and other systemic barriers are addressed. Since 2019, WHO EML has included nivolumab and pembrolizumab as first-line monotherapy for metastatic skin melanoma.^14^ However, the EML expert committee rejected the proposed inclusion of PD1/PD-L1 inhibitors for advanced non-small cell lung cancer (NSCLC) in 2019, 2021, and 2023.^14^, ^15^, ^16^ The committee acknowledged the efficacy and safety of these medicines as first-line monotherapies for advanced NSCLC, which demonstrate substantial benefits according to the European Society of Medical Oncology (ESMO)’s rating of at least 4 on its Magnitude of Clinical Benefit Scale.^17^ However, the committee expressed concerns about their costs and affordability in many country settings at the prevailing market price, noting the high global prevalence of NSCLC and “the opportunity costs of providing treatment with immune checkpoint inhibitors would be substantial for many health systems and would divert limited available resources from other public health programmes” (p. xix).^16^ In this context, understanding the budget impact of expanding access to immune checkpoint inhibitors is important to the broader discussion on identifying pathways and strategies that ensure these beneficial treatments are delivered cost-effectively, timely, and appropriately.

To this end, this paper presents a modelling study that quantified the financial impacts of expanding access to PD1/PD-L1 inhibitors as first-line monotherapy for advanced unresectable NSCLC in patients with wildtype epidermal growth factor receptor (EGFR) PD-L1 tumoural proportion score (TPS) ≥50%. The scope of this modelling study has three main rationales: (1) NSCLC has significant public health relevance especially in lower income countries; (2) multiple PD1/PD-L1 inhibitors (i.e., atezolizumab, cemiplimab, pembrolizumab) have been approved as first-line monotherapy, showing superior patient survival compared to chemotherapy, and offering a practical advantage for roll-out in lower income countries compared to combination treatments; and (3) molecular profiling of tumour cells for *EFGR* mutation status and PD-L1 expression can be used to identify patients most likely to benefit. As this is not an economic evaluation, the study did not assess the comparative cost-effectiveness of PD1/PD-L1 inhibitors for metastatic NSCLC, which could vary significantly by country contexts due to different potential alternative use of financial resources (i.e., opportunity cost).

## Methods

### Study design

**Fig. 1:**
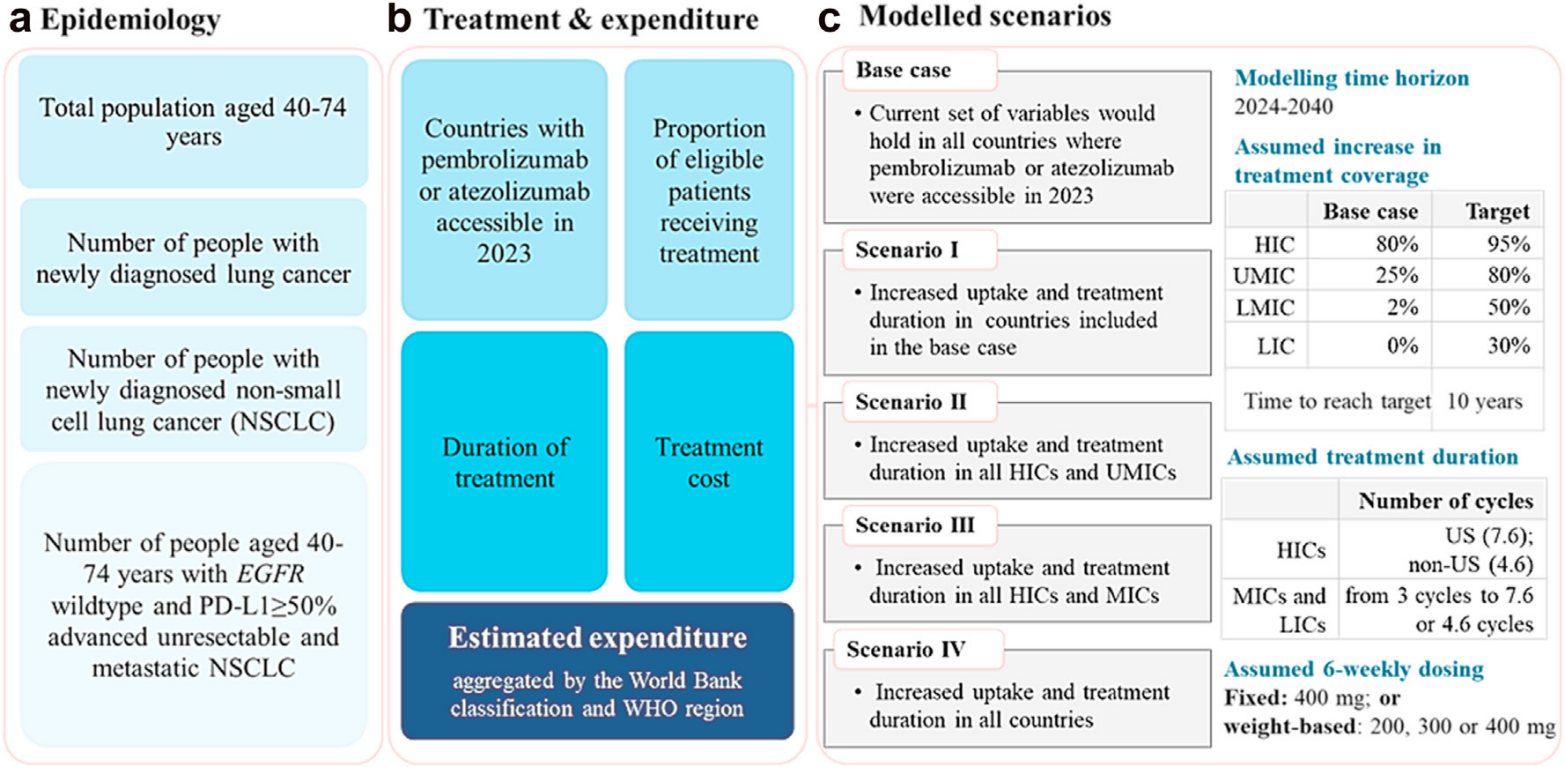
Model structure and scenarios. NSCLC = non-small cell lung cancer; EGFR = epidermal growth factor receptor; PD-L1 = Programmed death-ligand 1; HIC = high-income countries; MIC = Middle-income countries; UMIC = Upper-middle-income countries; LMIC = lower-income countries; LIC = low-income countries; WHO = World Health Organization. a: Epidemiology, b: Treatment & expenditure, c: Modelled scenarios.

A three-part deterministic model, shown in Fig. 1, was developed in Microsoft Excel to estimate the potential usage of PD1/PD-L1 inhibitors for treating advanced NSCLC and the associated financial costs, evaluated through repeated cross-sectional assessments from 2024 to 2040. The model was populated with data extracted from the best available sources identified through a targeted search of the literature, websites, and expert consultations in 2022–2025. Table 1 presents the input parameters along with corresponding explanations and assumptions. The complete list of references is provided in the Appendix.

**Table 1:** Input parameters.^a^

Parameter	Description
**Epidemiology**	
Country population aged 40–74 years	Numbers in 5-year age group, by country
Incidence of lung cancer	Rates in 5-year age group, by country
Proportion of NSCLC among lung cancer cases	Percentages by country Median = 87.9% (range = 76.9%–99.3%)
Proportion advanced NSCLC unresectable at presentation	HIC = 65.7%; UMIC = 85.8%; LMIC/LIC = 100%
Prevalence of *EGFR* mutation in NSCLC (for exclusion)	Percentages by country Median = 28.7% (range = 8.3%–51.1%)
Proportion PD-L1 TPS ≥ 50% among *EGFR-*wildtype tumours	25.3%
**Health service**	
Accessibility^c^ of PD1/PD-L1 inhibitors in 2023	41 HICs; 18 UMICs; 3 LMICs; 0 LICs
Proportion of patients receiving molecular tests^a^	HIC = 90%; UMIC = 56%; LMIC = 20%; LIC = 5%
Treatment rate with immune checkpoint inhibitors	**Base case:** HIC = 80%; UMIC = 25%; LMIC = 2%; LIC = 0% **Modelled**: HIC = 95%; UMIC = 80%; LMIC = 50%; LIC = 30%
Average person-months on treatment	**Base case:** US = 10.5; Non-US HIC^b^ = 7.4; MIC = 4.2; LIC = 4.2 **Modelled: all countries** = 10.5
Dosing	Standard fixed dosing: 400 mg every 6 weeks Weight-based dosing: 200 mg, 300 mg or 400 mg every 6 weeks^d^
**Cost**	
Ex-manufacturer price of pembrolizumab 100 mg/4 mL vial	**Base case and modelled scenarios assumed median price**: US$2478 **Sensitivity analysis**: US$1497 (lowest price from the sources)
Estimated average lifetime treatment cost per patient	**Base case** US = US$75,100; Non-US HICs and UMICs = US$53,000; LMIC/LICs = US$30,100 **Modelled scenarios:** *Standard fixed dosing*, with all patients receiving treatment for an average duration of 10.5 months (i.e., 7.6 of 6-weekly cycles) = US$75,100 *Weight-based dosing*, with all patients receiving treatment for an average duration of 10.5 months (i.e., 7.6 of 6-weekly cycles) = US$37,600 to US$75,100
Cost of single immunohistochemical tests for PD-L1 expression	Median = US$105 Range = US$49; US$217

NSCLC = non-small cell lung cancer; HIC = high-income countries; UMIC = upper-middle-income countries; LMIC = lower-middle-income countries; LIC = low-income countries.

a All sources are listed in Table A1 in the Appendix.

b Based on Japan and EU5 France, Germany, Italy, Spain, and UK.

c Accessibility was assessed using a composite score based on the availability and financial coverage of pembrolizumab or atezolizumab for lung cancer from ESMO's global survey of antineoplastic medicines.

d 200 mg every 6 week for individuals weighing <65 kg; 300 mg every 6 weeks for 65–90 kg; or 400 mg every 6 weeks for ≥90 kg.

To estimate the potential extent of use, the model employed an epidemiological approach to determine the number of people with NSCLC eligible for receiving PD1/PD-L1 inhibitors as monotherapy. This involved considering country population projection from 2024 to 2040, the incidence of advanced NSCLC that are unresectable or metastatic at clinical presentation, along with the tumour characteristics (i.e., PD-L1 TPS ≥50%). Patients with *EGFR*-mutated advanced NSCLC were excluded as the standard first-line treatment are *EGFR* tyrosine kinase inhibitors. The model restricted the age range to 40–74 years, encompassing most of the eligible population with a performance status sufficient to tolerate and therefore benefit from PD1/PD-L1 inhibitors. Using the epidemiological estimates, the model calculated the treatment uptake by factoring in health-system variables. First, the model considered accessibility of pembrolizumab or atezolizumab in WHO’s 194 Member States in 2023, according to a composite score derived from ESMO's global survey of antineoplastic medicines regarding availability and financial coverage for NSCLC (Appendix).^18^ Cemiplimab was not included in the ESMO survey, but its accessibility was likely lower than pembrolizumab and atezolizumab due to its later market introduction. The model then incorporated the proportion of eligible patients likely to receive treatment informed by real-world evidence where available or extrapolated according to the country’s corresponding World Bank’s country classifications by income level (i.e., high-, upper-middle-, lower-middle-, and low-income countries; abbreviated respectively as HIC, UMIC, LMIC, and LIC hereafter) when data was absent. The model assumed that countries would maintain their 2024 World Bank income classification until 2040.

### Modelled scenarios

The model analysed the base-case scenario alongside four predefined alternatives to forecast treatment adoption and its financial impacts from 2024 to 2040. The base-case scenario projected the continuation of real-world treatment rates and treatment durations in countries where the PD1/PD-L1 inhibitors were accessible and their associated financial implications. In contrast, the four alternative scenarios evaluated the financial impacts of achieving predefined target uptake rates and uniform duration of treatments as observed in high-income countries (HICs) (Table 1). These scenarios modelled the expansion of treatment to reach target uptake rates across four groups of countries: (1) countries where pembrolizumab or atezolizumab were accessible in 2023 as in the base case; (2) all HICs and UMICs; (3) all HICs and MICs; and (4) all countries. The target coverage rates, ranging from 30% to 95%, were expected to increase from baseline levels of 0% in LICs to 80% in HICs over 10 years, with corresponding annualized growth rates between 1.4% and 4.5%. The model estimated the average person-months on treatment based on real-world data where possible (Appendix) to avoid underestimation of the usage based on the reported median time on treatment (4.2–6.7 months)^19^, ^20^, ^21^ because of a significant proportion of patients remaining on treatment at 2 years. The model assumed optimistically that MICs and LICs would extend treatment duration (3–4.2 months) to the average person-months on treatment in HICs (10.5 months), with treatment discontinuation occurring after 2 years, in line with current recommendations.^22^ The model considered two 6-week dosing regimens for pembrolizumab: standard recommended dose of 400 mg, or a hybrid dosing regimen that combines weight-based and flat dosing of 200, 300 or 400 mg. The weight-based dosing regimen has been implemented in countries, including Canada,^23^ the Netherlands,^5^ England,^11^ and Singapore.^6^ Although not considered in this study, alternative dosing regimens have also been proposed for atezolizumab and nivolumab.^5^^,^^24^

### Expenditure analysis and reporting

The cost assessment was conducted from a healthcare system perspective. The projected costs of using PD1/PD-L1 inhibitor monotherapies in eligible patients were calculated based on the ex-manufacturer price of pembrolizumab listed on the government websites of ten countries (Table 1 and Appendix), as well as the anticipated dosage and duration of use, informed by real-world clinical practice described in the previous section. To simplify the analysis and minimize uncertainty, the study excluded mark-ups and taxes in the supply and distribution chain, and healthcare service costs related to the administration of PD1/PD-L1 inhibitors and follow-up care, as these costs can vary significantly across countries and in-country healthcare settings. Additionally, the model assumed that standard first-line treatments for advanced NSCLC, such as platinum-based doublet chemotherapy (e.g., gemcitabine-cisplatin, pemetrexed-cisplatin, paclitaxel-carboplatin), would be replaced by PD1/PD-L1 inhibitor monotherapies in eligible patients, as chemotherapy results in shorter survival. The cost of PD-L1 testing was estimated based on the number of advanced unresectable NSCLC cases and real-world testing rates. This model excluded the cost of *EGFR* testing, as it was attributed to assessing the suitability of *EGFR* tyrosine kinase inhibitors rather than PD1/PD-L1 inhibitors.

The model analysed the financial costs of PD1/PD-L1 inhibitors for treating advanced NSCLC and PD-L1 testing by individual countries. The estimates were aggregated by the four World Bank’s country income classifications in 2025 and the six WHO regions for Africa (AFR), the Americas (AMR), Eastern Mediterranean (EMR), Europe (EUR), South-East Asia (SEAR), and the Western Pacific (WPR). Cost estimates were expressed in United States dollars (US$) in nominal value without discounting, and rounded to the nearest thousands to reflect the assumed level of precision. The analysis examined the incremental difference between base-case and the modelled scenarios to estimate the additional costs required to achieve the target treatment coverage rates. The expenditure estimates were expressed as proportions of the total pharmaceutical expenditure, calculated from reported total health expenditure and the proportions attributed to pharmaceuticals (Appendix).

### Uncertainty and model validation

The model study considered two sources of uncertainty that could impact the financial estimates that were excluded from the primary analysis because a lack of more reliable estimates has limited their usefulness from a modelling perspective. First, the model applied the median ex-manufacturer list price sourced from a set of ten countries. However, list prices may not reflect the final price due to common and often substantial discounts, rebates, or other incentives, which are typically confidential. Notwithstanding, some list prices suggest the potential magnitude of discrepancies between the list and the net prices. For example, the price of a 4 mL vial of pembrolizumab 100 mg listed in the Japanese Government National Health Insurance Drug Price Standard (JP¥214,498 ≈ US$1500)^25^ was the lowest among the sources consulted. This price is close to the final price, as it is based on the weighted average of actual transaction prices from wholesalers to medical institutions or pharmacies, as determined by a survey conducted by Japan’s Ministry of Health, Labour and Welfare.^26^^,^^27^ While market conditions would vary by country, the model tested the potential financial impacts of using an assumed net transaction price of US$1500 instead of US$2478. Second, the primary patents of several immune checkpoint inhibitors will end within the modelling period, including pembrolizumab in 2028 and atezolizumab in 2029. Competition from biosimilar products following the conclusion of patent term or exclusivity period can reduce price significantly, potentially improving access in countries where affordability has previously been a barrier. However, the magnitude of price reduction and market penetration of biosimilar products can vary significantly by contexts, influenced by factors such as secondary patents and exclusivity period, regulatory approval processes, manufacturing and supply chain issues, individual market characteristics, and legal disputes.^28^, ^29^, ^30^, ^31^, ^32^ This study considered this factor qualitatively and presented in the discussion section. The model outputs were checked for technical validity, face validity and predictive validity (Appendix).

Finally, emerging proof-of-concept evidence, such as the interim analysis of the NVALT-30 trial,^33^ suggests that low-dose immunotherapies could be as effective as standard doses for treating advanced metastatic NSCLC. However, further evidence is necessary before any clinical recommendations can be made regarding potential schedule changes, such as adopting a 200 mg dose of pembrolizumab every 6 weeks. To this point, a sensitivity analysis was conducted to assess the potential increase in the number of patients that could be treated within the projected expenditure under the base case and Scenario 4, if the proposed low-dose schedule were to become the standard of care.

### Role of the funding source

The funding source had no role in the study’s design, data collection, analysis, interpretation, or reporting.

## Results

From 2024 to 2040, the model projected an increase in the number of people aged 40–74 years with NSCLC, rising from 1.49 million to 1.85 million. The growth rates were expected to be highest in LMICs (+48%) and LICs (+65%). However, the absolute increase in number of cases was higher in UMIC (973,000) and HIC (460,000) in 2040, driven by population size, older age composition, and higher lung cancer incidence rates. Among this population, approximately 13.5% (200,000–250,000 individuals) were expected to have advanced NSCLC with *EGFR*-wildtype PD-L1 TPS≥50% tumors that were unresectable or metastatic at diagnosis, and eligible to receive first-line PD1/PD-L1 monotherapy. If current treatment rates continue in countries where the PD1/PD-L1 inhibitors were accessible in 2023 (i.e., base case), only about one-fifth of the eligible population (45,100–48,300 individuals) would receive treatment with PD1/PD-L1 inhibitors during the modeling period. The disparity in access would persist, with HICs and UMICs accounting for nearly the entirety of the global population receiving treatment. For this population, the base-case model estimated the stable global expenditure, growing from US$2605 million to US$2773 million by 2040 (Table 2).

**Table 2:** Estimated number of people aged 40–74 years treated by PD1/PD-L1 inhibitor monotherapies for advanced NSCLC with *EGFR*-wildtype PD-L1 TPS≥ 50% tumours and the associated financial costs for medicines and diagnostics, 2024–2040.

	Base case		Scenario 1		Scenario 2		Scenario 3		Scenario 4	
Coverage assumption	Continuation of existing coverage rates		Uptake rates increase to meet target coverage in 10 years: HIC (80%–95%), UMIC (25%–80%), LMIC (2%–50%), LIC (0%–30%)							
Scope of expansion	Countries with access to PD1/PD-L1 inhibitors		Same countries as in the base case		All HICs and UMICs		All HICs and MICs		All countries	
Year	2024	2040	2024	2040	2024	2040	2024	2040	2024	2040
**By World Bank country income classification**										
Number of people treated										
High income	39,800	42,000	40,500	49,900	40,600	50,200	40,600	50,200	40,600	50,200
Upper-middle income	5200	6100	6100	19,400	11,300	106,800	11,300	106,800	11,300	106,800
Lower-middle income	100	200	300	4300	300	4300	1700	28,500	1700	28,500
Low income	0	0	0	0	0	0	0	0	100	2200
Total	45,100	48,300	46,900	73,600	52,200	161,200	53,600	185,400	53,700	187,600
Cost of PD1/PD-L1 inhibitors (million US$)										
Fixed dosing										
High income	$2329	$2447	$3044	$3747	$3045	$3767	$3045	$3767	$3045	$3767
Upper-middle income	$273	$321	$457	$1456	$849	$8017	$849	$8017	$849	$8017
Lower-middle income	$3.3	$5.1	$24	$321	$24	$321	$128	$2140	$128	$2140
Low income	$0.0	$0.0	$0.0	$0.0	$0.0	$0.0	$0.0	$0.0	$8.7	$162
Total	$2605	$2773	$3526	$5525	$3919	$12,105	$4022	$13,924	$4031	$14,087
Weight-based dosing										
High income	$2329	$2447	$2529	$3110	$2530	$3128	$2530	$3128	$2530	$3128
Upper-middle income	$273	$321	$359	$1145	$564	$4595	$564	$4595	$564	$4595
Lower-middle income	$3.3	$5.1	$15	$202	$15	$202	$75	$1249	$75	$1249
Low income	$0.0	$0.0	$0.0	$0.0	$0.0	$0.0	$0.0	$0.0	$5.9	$109
Total	$2605	$2773	$2903	$4457	$3109	$7924	$3169	$8972	$3174	$9080
Cost of diagnostic tests (million US$)										
High income	$27	$29	$27	$29	$27	$29	$27	$29	$27	$29
Upper-middle income	$7	$8	$7	$8	$46	$57	$46	$57	$46	$57
Lower-middle income	$1	$1	$1	$1	$1	$1	$5	$8	$5	$8
Low income	$0	$0	$0	$0	$0	$0	$0	$0	$0	$0
Total	$35	$39	$35	$39	$74	$88	$78	$95	$79	$95
**By WHO region**										
Number of people treated										
Africa	0	0	0	0	100	1500	200	3900	200	5200
Americas	16,700	18,000	17,300	26,700	17,500	29,100	17,500	29,500	17,500	29,500
Eastern Mediterranean	400	900	600	4400	600	4900	700	6500	700	7000
Europe	22,700	23,600	23,600	34,400	23,800	38,500	23,900	40,100	23,900	40,100
Southeast Asia	0	0	0	0	100	1800	1000	17,100	1000	17,500
Western Pacific	5300	5800	5400	8100	10,100	85,500	10,300	88,200	10,300	88,200
Total	45,100	48,300	46,900	73,600	52,200	161,200	53,600	185,400	53,700	187,600
Cost of PD1/PD-L1 inhibitors (million US$)										
Fixed dosing										
Africa	$0.0	$0.0	$0.0	$0.0	$5.4	$111	$14.0	$296	$18.6	$389
Americas	$1147	$1220	$1300	$2004	$1311	$2187	$1312	$2217	$1312	$2217
Eastern Mediterranean	$21.2	$44	$46	$334	$47	$365	$54	$489	$56	$526
Europe	$1162	$1209	$1772	$2581	$1789	$2888	$1798	$3010	$1798	$3010
Southeast Asia	$0.0	$0.0	$0.0	$0.0	$9	$132	$74	$1284	$76	$1317
Western Pacific	$275	$300	$407	$606	$757	$6422	$770	$6628	$770	$6628
Total	$2605	$2773	$3526	$5525	$3919	$12,105	$4022	$13,924	$4031	$14,087
Weight-based dosing										
Africa	$0.0	$0.0	$0.0	$0.0	$4.0	$82	$10.4	$220	$13.5	$281
Americas	$1147	$1220	$1234	$1835	$1242	$1975	$1243	$1998	$1243	$1998
Eastern Mediterranean	$21.2	$44	$32	$211	$33	$235	$38	$327	$39	$350
Europe	$1162	$1209	$1401	$2041	$1413	$2271	$1420	$2362	$1420	$2362
Southeast Asia	$0.0	$0.0	$0	$0	$5	$66	$38	$665	$40	$689
Western Pacific	$275	$300	$237	$370	$413	$3295	$419	$3400	$419	$3400
Total	$2605	$2773	$2903	$4457	$3109	$7924	$3169	$8972	$3174	$9080
Cost of diagnostic tests (million US$)										
Africa	$0.00	$0.00	$0.00	$0.00	$0.43	$0.66	$0.82	$1.36	$0.89	$1.49
Americas	$4.8	$6.0	$4.8	$6.0	$5.8	$7.4	$5.8	$7.5	$5.8	$7.5
Eastern Mediterranean	$1.0	$1.8	$1.0	$1.8	$1.1	$2.0	$1.4	$2.4	$1.4	$2.5
Europe	$23	$24	$23	$24	$24	$26	$25	$26	$25	$26
Southeast Asia	$0.0	$0.0	$0.0	$0.0	$1.0	$1.1	$3.8	$5.2	$3.8	$5.2
Western Pacific	$6.5	$7.2	$6.5	$7.2	$41	$51	$42	$52	$42	$52
Total	$35	$39	$35	$39	$74	$88	$78	$95	$79	$95

NOTE: Numbers may not add up due to rounding.

TPS = PD-L1 tumoural proportion score; HIC = High-income countries; UMIC = Upper-middle-income countries; LMIC = Lower-middle-income countries; LIC = Low-income countries; US$ = United States dollar.

Modelled scenarios suggest that expanding PD1/PD-L1 inhibitor monotherapies to the assumed coverage rates of up to 30% in LICs and 95% in HICs over the next 10 years could increase the number of people treated globally by as much as 139,300 by 2040, compared to the base case (Table 2). This would raise the global treatment coverage rate up to 75% assuming expanded access across all countries in scenario 4 (Table 2). A large proportion of this projected increase in the number of people treated with PD1/PD-L1 inhibitor monotherapies by 2040 would occur in UMICs (+100,700), compared to LMICs (+28,300), HICs (+8200) and LICs (+2200) (Table 2). Geographically, the model predicts that WPR will see the largest increase in the number of people treated by 2040, with an additional 82,400 people treated, far outpacing all other WHO regions, where the increases would range from 5200 to 17,500 (Table 2).

**Fig. 2:**
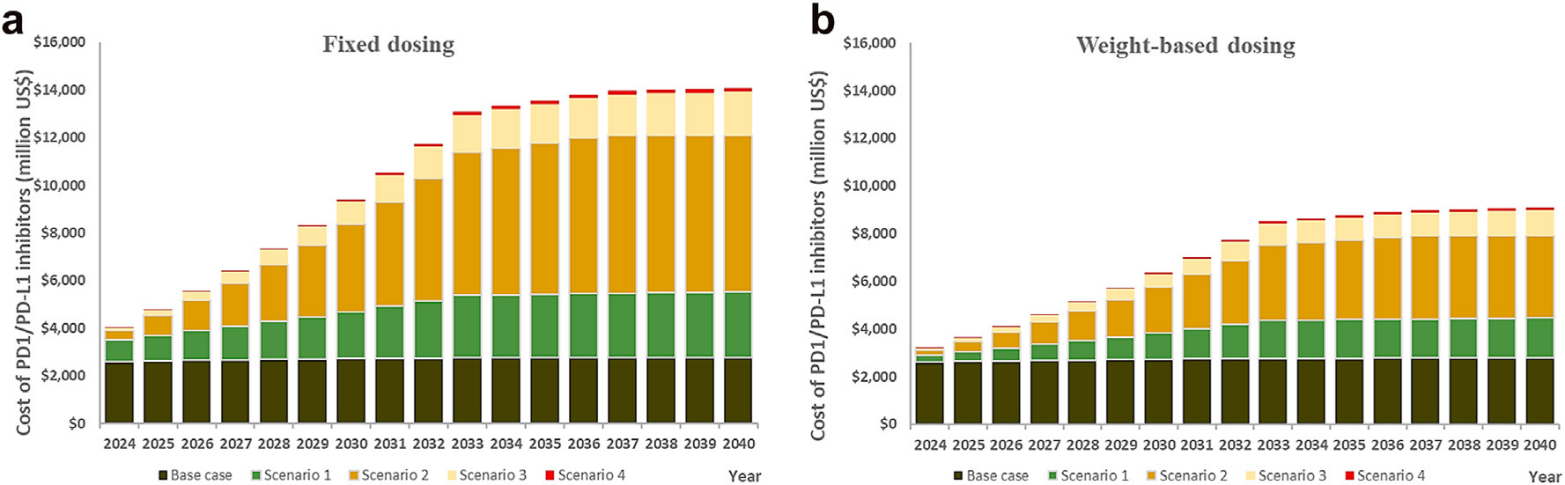
The costs associated with PD1/PD-L1 inhibitor monotherapies, by year and scenario. a: Fixed dosing, b: Weight-based dosing.

Assuming fixed dosing at 400 mg pembrolizumab every 6 weeks, by 2040, the model projected a potential four-fold increase, or US$11,313 million, because of modelled access expansion compared to the base case, bringing the global expenditure on PD1/PD-L1 inhibitor monotherapies for the modelled population to US$14,087 million (Fig. 2). The largest expenditure increases by 2040 were observed in UMICs (+US$7696 million) and WPR (+US$6327 million), reflecting the high rates of modelled expansion in access in UMICs (Table 2). These were followed by EUR (+US$1802 million), SEAR (+US$1317 million), AMR (+US$997 million), EMR (+US$481 million), and AFR (+US$389 million). Assuming weight-based dosing, the model projected up to 227% increase (+US$6307 million) in expenditure, lowering the projected global expenditure on PD1/PD-L1 inhibitor monotherapies for the modelled population to US$9080 million (Fig. 2 and Table 2). By 2040, the global cost of immunohistochemical tests to assess PD-L1 expression was estimated to range from US$39 million (base case) to US$95 million (scenario 4) in 2040, representing less than 1% of the total estimated expenditure.

**Fig. 3:**
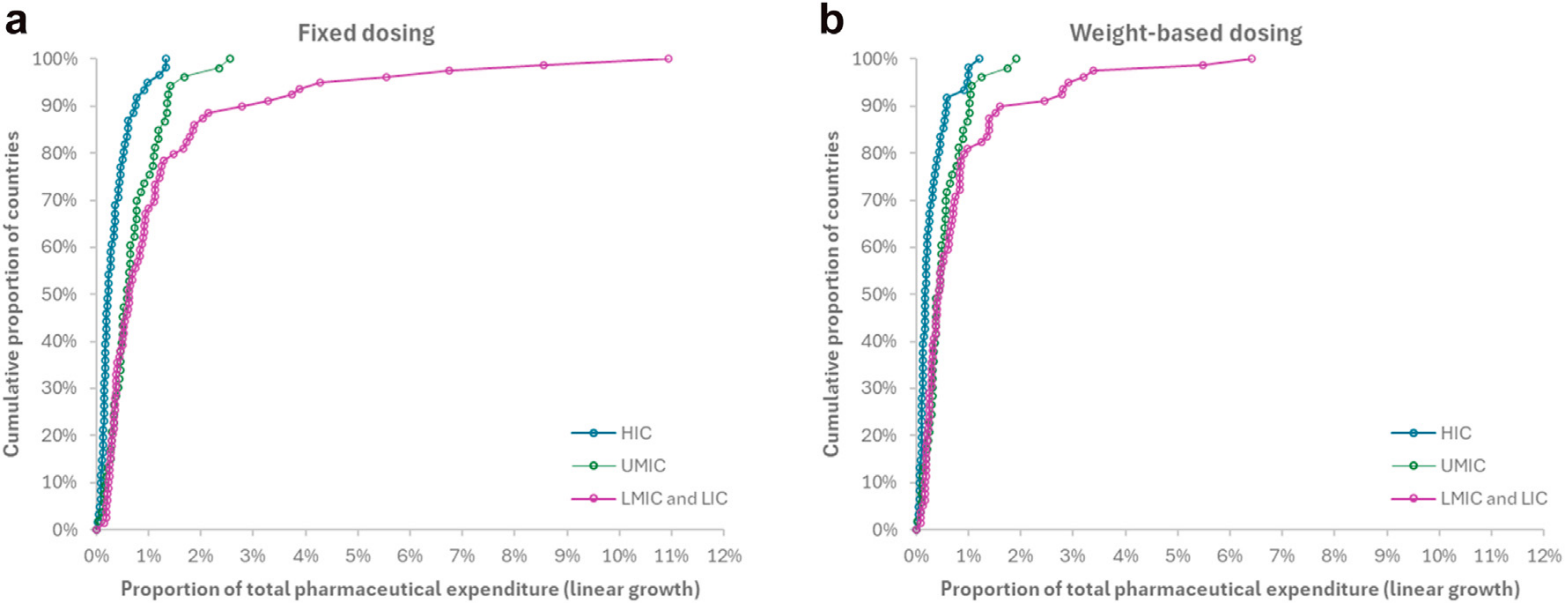
Proportions of total pharmaceutical expenditure required to meet the estimated costs of PD1/PD-L1 inhibitor monotherapies. HIC = high-income countries; MIC = Middle-income countries; UMIC = Upper-middle-income countries; LMIC = lower-income countries; LIC = low-income countries; WHO = World Health Organization. Note: Each circle represents a country. a: Fixed dosing, b: Weight-based dosing.

For at least 25% of MICs and LICs, expanding access to the recommended fixed-dose PD1/PD-L1 inhibitor monotherapies for advanced NSCLC would result in significant expenditure, exceeding 1% to as much as 11% of the projected total pharmaceutical expenditure from 2024 to 2040 (Fig. 3a). Adopting weight-based dosing would lower the percentage of MICs and LICs needing to allocate more than 1% of the projected total pharmaceutical expenditure for treating advanced NSCLC with PD1/PD-L1 inhibitor monotherapies to around 13% (Fig. 3b).

A one-way sensitivity analysis using an assumed price of US$1500 for PD1/PD-L1 inhibitors (c.f. US$2478 in the primary analysis) while holding all other parameters constant found a corresponding 40% reduction in the projected expenditure. At this price, the expenditure by 2040 would reduce to US$8527 million with fixed dosing or US$5497 million with weight-based dosing, assuming expanded access to all countries under the conditions outlined in scenario 4. The lower costs associated with weight-based dosing suggest increased efficiency, potentially allowing more patients to benefit from having access. Further price discounts or rebates would lead to proportional reductions in the estimated expenditure presented in Table 2. Assuming a fixed low-dose pembrolizumab regimen of 200 mg every 6 weeks, the number of patients treated would increase by 53% in the base case and by 29% in Scenario 4, compared to the modelled weight-based dosing scenario. As expected, this low-dose schedule would double the projected number of patients treated compared to the standard fixed on-label 400 mg every 6 weeks regimen.

## Discussion

The vast disparity in global access to immune checkpoint inhibitors has overshadowed the scientific and commercial successes of the past decade. With the established efficacy and safety when used by trained professionals, using PD1/PD-L1 inhibitors to treat patients with advanced NSCLC can address an important public health problem and align with the principles of essential medicines. However, high treatment cost has undermined their cost-effectiveness, created financial burdens, and exacerbated inequities within and between countries.

This study estimated the costs associated with expanding access to PD1/PD-L1 inhibitors as first-line monotherapy for individuals aged 40–74 years with *EGFR*-wildtype PD-L1 TPS ≥ 50% unresectable or metastatic NSCLC. The model's results indicate that expanding access to target coverage rates—30% in LICs, 50% in LMICs, 80% in UMICs, and 95% in HICs—over the next 10 years could lead to a nearly fourfold increase in the number of patients treated, compared to the baseline scenario. This would raise global treatment coverage for eligible patients aged 40–74 years from an estimated 22% in 2024 (which was concentrated in HICs) to as high as 75% globally, with a particularly significant increase in middle-income countries.

Based on the median list price and real-world treatment durations, the projected cost of expanding access to PD1/PD-L1 inhibitor monotherapies by 2040 could reach up to US$14,087 million, assuming treatment with a fixed dose of 400 mg pembrolizumab every 6 weeks for an average of 10.5 months (i.e., US$75,100 per patient). Adopting weight-based dosing, for US$37,600–US$75,100 per patient, would lower the estimated expenditure to US$9080 million. A price of US$1500 per 100 mg/4 mL vial of pembrolizumab might be indicative of the possible price after discounts and rebates. In this case, the projected expenditure by 2040 would decrease by 40%, falling to US$8527 million or US$5497 million, depending on fixed or weight-based dosing. The cost of PD-L1 expression assessment would be less than 1% of the total estimated cost, reflecting the lower price of a single immunohistochemical test (US$105) compared to the total costs of PD1/PD-L1 inhibitors.

At the current price, the estimated financial resources required to progressively expand access to PD1/PD-L1 inhibitors as monotherapies for advanced NSCLC in all countries over 10 years are significant compared to the current and projected pharmaceutical expenditure, casting doubt on the feasibility of achieving equitable access in lower-income countries. Financial resources allocated to the use of PD1/PD-L1 inhibitors in lower-income countries must be carefully assessed for cost-effectiveness and weighed against the opportunity costs arising from the potential benefits lost from alternative uses (e.g., an estimated additional US$20 billion per year required to implement an essential package of cancer control interventions^34^). To further illustrate the potential trade-offs, based on an absolute risk reduction of 12.1% reported in the KEYNOTE-042 study,^35^ eight patients with high PD-L1 expression would need to be treated with pembrolizumab monotherapy to keep one patient alive at five years. These trade-offs would increase with higher prices, and decision-makers would need to weigh them against their value judgments and from other health needs.

Market competition can facilitate price reductions and broaden access to medicines. The WHO guidelines strongly recommend promoting the use of quality-assured generic and biosimilar medicines, not only because they are priced lower than originator products but also due to the competitive pricing they generate.^36^ Studies have demonstrated a significant reduction in the prices of both referenced biologic products and biosimilar medicines following the introduction of biosimilars, with sustained price declines in subsequent years.^28^^,^^30^ Such price reductions and improvements in access should be expected for PD1/PD-L1 inhibitor biosimilars upon their entry, at least once the primary patents expire (e.g., pembrolizumab in June 2028 and atezolizumab in December 2029^37^). However, policymakers must be mindful of the detrimental effects of complex patent clusters (‘patent thickets’) and strategies designed to extend market exclusivity (‘evergreening’), which seek to delay the entry of biosimilar medicines and prolong profitability at the expense of public health benefits.^38^^,^^39^ Policymakers should proactively address and prevent such practices, while implementing regulatory and administrative measures that facilitate the early market entry of biosimilar medicines and maximize the uptake of biosimilars (e.g., interchangeability policies^40^).

To promote price competition among on-patent products, policymakers and the scientific community may consider several measures to improve spending efficiency. These include exploring optimal dosing regimens and shorter treatment duration,^5^ clarifying class therapeutic effects and interchangeability of PD1/PD-L1 inhibitors,^7^ implementing therapeutic group tendering (e.g., Denmark^41^), and conducting efficient pooled procurements backed by adequate, stable, and predictable financing to enhance purchasing power.^42^ When efforts to negotiate affordable prices to enhance access prove insufficient, countries may consider utilizing voluntary licensing agreements, including those negotiated through the Medicines Patent Pool. Where necessary and appropriate, countries may explore using flexibilities under the World Trade Organization's Trade-Related Aspects of Intellectual Property Rights to address patent-related barriers to access.

The discussion on fair return concerning medicine pricing should continue. Policies should be designed to ensure that companies receive a fair and reasonable financial return on their investments during the legally protected monopoly period, on the condition that it also protects consumers from excessively high prices that prevent the full societal benefits of innovations from being realized. Research has shown that, through high prices, cancer medicines have yielded returns for originator companies far exceeding the likely research and development costs.^43^ It is important to challenge the narrative that high prices are the sole necessary mechanism to incentivize innovation. Innovations like PD1/PD-L1 inhibitors should benefit society, rather than perpetuating or exacerbating inequities.

There are several limitations to this analysis. First, the costs of managing immune-related adverse events and other health services associated with using PD1/PD-L1 inhibitors have not been quantified. Evidence indicates that severe immune-related adverse events can occur in 5%–10% of patients.^44^ These adverse events typically occur within the first three treatment months but can have delayed onset long after treatment discontinuation. Identifying and managing these adverse events often require specialized clinical expertise, and may occasionally necessitate costly therapeutics (e.g., infliximab or immunoglobulins).^44^ Depending on the health system context, the need for specialized human resources, healthcare services, diagnostics, and treatments can significantly increase the financial costs of expanding access to PD1/PD-L1 inhibitors for advanced NSCLC.

Second, this study focused on using PD1/PD-L1 inhibitors as first-line monotherapies in the advanced NSCLC population most likely to benefit from the treatment because of practical and financial considerations. However, PD1/PD-L1 inhibitors have also shown to be beneficial for TPS<50% *EGFR*-wildtype NSCLC, as combination therapy, or used as a later-line treatment^8^ and in an adjuvant setting. In clinical practice, these medicines are also used for various malignancies beyond advanced NSCLC, including haematological cancers. In health systems where clinical guidelines and expenditure controls are not rigorously enforced, using PD1/PD-L1 inhibitors for non-approved or off-label indications can add further financial burden and reduce overall spending efficiency.

In conclusion, expanding access to PD1/PD-L1 inhibitors as first-line monotherapy for individuals aged 40–74 years with *EGFR*-wildtype PD-L1 TPS≥50% advanced NSCLC over the next 10 years to target rates of 30%–95% would result in up to four times more eligible patients receiving and benefiting from the treatment, with the most notable expansion in middle-income countries. However, at the current price, this expanded access would incur substantial costs compared to the base case and total pharmaceutical expenditure. The projected expenditure on PD1/PD-L1 inhibitors must be carefully assessed for cost-effectiveness and weighed against the opportunity costs, especially in lower-income countries. In seeking to expand affordable access to PD1/PD-L1 inhibitors for advanced NSCLC, policymakers should work with the stakeholders to negotiate lower prices, implement measures to improve spending efficiency (e.g., optimizing dose and treatment duration) and enhance health system capacity, including ensuring appropriate use and its readiness for introducing more affordable biosimilar medicines.

## Contributors

All authors confirm having full access to all the data in the study and accept responsibility to submit for publication. All authors confirm meeting all four criteria for authorship in the ICMJE Recommendations. Kiu Tay-Teo led the conceptualization of this study, data curation, formal analysis, methodology, project administration, resources, and writing. The lead author Kiu Tay-Teo takes responsibility for the integrity of the underlying data and the accuracy of the data analysis and assumptions, which were contributed and verified by all other authors. All other authors contributed to reviewing the study scope, design, methodology, analysis, and interpretation, as well as editing the original and revised drafts. All authors approved the final version of the manuscript.

## Data sharing statement

No primary data was obtained for this analysis. The model utilises secondary data with all sources available from published literature, which have been reported in the manuscript or Appendix.

## Declaration of interests

The authors alone are responsible for the views expressed in this article and they do not necessarily represent the views, decisions or policies of the institutions with which they are affiliated.

Elisabeth G.E. de Vries declares institutional financial support outside the submitted work from various pharmaceutical companies and a National Cancer Institute’s clinical trials cooperative group: for clinical trials or contracted research (Amgen, Genentech, Roche, Servier, Regeneron, Crescendo Biologics and Bayer), consultancy (Crescendo Biologics), participation on a Data Safety Monitoring Board or Advisory Board (NSABP or National Surgical Adjuvant Breast and Bowel Project, Daiichi Sankyo). Elisabeth G.E. de Vries declares non-financial interest as a member of the European Society for Medical Oncology’s Magnitude of Clinical Benefit Scale (ESMO-MCBS) Working Group and Cancer Medicines Committee.

All other authors declare no competing interests.
