# Supplementary material for: Modelling the potential financial impacts of expanding access to immune checkpoint inhibitors as monotherapy for treating advanced non-small cell lung cancer
Source: eClinicalMedicine. 2025 May 24;84:103261. doi: 10.1016/j.eclinm.2025.103261 (PMC12159930; doi:10.1016/j.eclinm.2025.103261)
Supplement: Appendix [file mmc1.docx]

**Appendix**

Table A1: Input parameters, sources and notes

| **Parameter** | **Value** | **Source and notes** |
| --- | --- | --- |
| **Epidemiology** |  |  |
| Country population aged 40–74 years | Numbers by 5-year age group and country | UN Department of Economic and Social Affairs^1^; values in 5-year age group and standard projection to 2040 |
| Incidence of lung cancer | Rates by 5-year age group and country | IHME Global Health Data Exchange^2^, which included cancers of the trachea and bronchus |
| Proportion of NSCLC among lung cancer cases | Median = 87.9% (range =76.9%–99.3%) | Derived based on the proportion of small-cell lung cancers reported by Zhang et al^3^ (Appendix Table S3) |
| Proportion advanced NSCLC unresectable at presentation | HIC = 65.7%; UMIC= 85.8%; LMIC/LIC = 100% | Stage IIIb and IV NSCLC estimated or assumed based on Morgensztern et al^4^; Casal-Mouriño et al^5^; Nwagbara et al^6^; de Lima et al^7^; resectability of stage III NSCLC was estimated based on Jazieh et al^8^ |
| Prevalence of *EGFR* mutation in NSCLC (for exclusion) | Median =28.7% (Range =8.3%–51.1%) | Arrieta et al^9^; Van Christ Manirakiza et al^10^; Melosky et al^11^; Leduc et al^12^; Rennert et al^13^; Boustany et al^14^; Noronha et al^15^ Benbrahim et al^16^ Martin et al^17^; Aye et al^18^; Yessentayeva et al^19^; Chan et al^20^; Kumari et al^21^; Palacio et al^22^; Yatabe et al^23^; |
| Proportion PD-L1 TPS ≥50% among *EGFR-*wildtype tumours | 25.3% | Dietel et al^24^: calculated from Table 1 (319/1260). The study found that the overall prevalence rates of PD-L1 TPS ≥50% were comparable across Europe (22%); Asia Pacific (22%); the Americas (21%); and other countries (24%). Parra-Medina et al^25^ was also referred to for validation. |
| **Health service** |  |  |
| Accessibility^§^ of PD1/PD-L1 inhibitors in 2023 | 41 HICs; 18 UMICs; 3 LMICs; 0 LICs | ESMO Study on availability and financial coverage of antineoplastic medicines^26^ |
| Proportion of patients receiving molecular tests† | 20% to 95% | Assumed based on testing rates of genetic mutations or tumour biomarkers reported in Smeltzer et al^27^; Kerr et al^28^; Chambers et al^29^; Martin et al^17^; How et al^30^; Lee et al^31^ |
| Treatment rate with immune checkpoint inhibitors | HIC= 80%; UMIC= 25%; LMIC=2%; LIC=0% | Assumed based on Carroll et al^32^; How et al^30^ ; Leung et al^33^; Noronha et al^34^; Griesinger et al^35^ |
| Average person-months on treatment | US= 10.5; Non-US HIC‡ =7.4; MIC/LIC= 4.2 | Derived from Velcheti et al^36^; Slowley et al^37^; Abraham et al^38^; Goto et al^39,40^ |
| Standard fixed dosing | 400mg every 6 weeks | Product information^41^ |
| Weight-based dosing | 200 mg, 300 mg or 400mg every 6 weeks^ | Hybrid dosing, as used in the Netherlands^42^ and is similar to the dosing in Singapore^43^, dose-banding in NHS England^44^, and weight based dosing in Canada^45^. Weight assumptions are based on Bonomi et al^46^ and NCD Risk Factor Collaboration^47^, without assuming future changes in body weight during the modelling time horizon. |
| **Cost** |  |  |
| Ex-manufacturer price of pembrolizumab 100mg/4 ML vial | Median= US$2,478 (range= US $1,497; US $2,788) | Assumed the median list price from Australia^48^, Belgium^49^, Brazil^50^, Bulgaria^51^, Chile^52^, India^53^, Japan^54^, Greece^55^, South Africa^56^, Türkiye^57^ |
| Estimated average lifetime treatment cost per patient for standard fixed dosing | **Base case**  US= US$75,100  Non-US HICs and UMICs = US$53,000; LMIC/LICs= US$30,100  **Modelled case**  US= US$75,100 | Calculated based on fixed dosing of 400mg every 6 weeks and an average person-months on treatment up to a maximum of 2 years |
| Estimated average lifetime treatment cost per patient for weight-based dosing | **Modelled case**  US$37,600 to US$75,100 | Calculated based on estimated/assumed unit price, dose based on median weights, and average time on treatment up to a maximum of 2 years |
| Cost of molecular test (Median, range) | US$105  (US$49; US$217) | Cost of single immunohistochemical test: Canada^58^; Australia^59^, India^60^, China (Hong Kong)^61^, China and US^62^ |

†Of the 64 countries responded to IASLC survey in 2019, 9 (14%) countries did not perform PD-L1 test for either clinical or research purposes^63^;

‡Based on Japan and EU5 France, Germany, Italy, Spain, and the United Kingdom

§Accessibility was assessed using a composite score based on the availability and financial coverage of pembrolizumab or atezolizumab for lung cancer from ESMO's global survey of antineoplastic medicines (appendix)

^200 mg every 6 weeks for individuals weighing <65 kg; 300 mg every 6 weeks for 65–90 kg; or 400 mg every 6 weeks for ≥90 kg.

Table A2: Country accessibility to pembrolizumab and atezolizumab for lung cancer based on ESMO's global survey of antineoplastic medicines^64^

| **Country** | **Financial coverage**^†^ | | **Availability**^‡^ | | **Combined score**^§^ | | | **Accessibility status^** |
| --- | --- | --- | --- | --- | --- | --- | --- | --- |
|  | Pembro | Atezo | Pembro | Atezo | | Pembro | Atezo |  |
| Afghanistan | 0 | 0 | 1 | 0.25 | | 0 | 0 | 0 |
| Albania | 0 | 0 | 0 | 0.25 | | 0 | 0 | 0 |
| Algeria | #N/A | #N/A | #N/A | 1 | | #N/A | #N/A | - |
| Argentina | 1 | 1 | 1 | 1 | | 1 | 1 | 1 |
| Armenia | 0 | 0 | 1 | 1 | | 0 | 0 | 0 |
| Australia | 1 | 1 | 1 | 1 | | 1 | 1 | 1 |
| Austria | 1 | 1 | 1 | 1 | | 1 | 1 | 1 |
| Azerbaijan | 0 | 0 | 0.75 | 0.25 | | 0 | 0 | 0 |
| Bangladesh | 0 | 0 | 1 | 0.5 | | 0 | 0 | 0 |
| Belarus | 0 | 1 | 0.25 | 0.5 | | 0 | 0.5 | 1 |
| Belgium | 1 | 1 | 1 | 1 | | 1 | 1 | 1 |
| Bolivia | 0 | 0 | 0.5 | 0.25 | | 0 | 0 | 0 |
| Bosnia and Herzegovina | 1 | 1 | 0.75 | 0.75 | | 0.75 | 0.75 | 1 |
| Botswana | 0 | #N/A | #N/A | #N/A | | #N/A | #N/A | - |
| Brazil | 1 | 1 | 1 | 1 | | 1 | 1 | 1 |
| Bulgaria | 1 | 1 | 1 | 1 | | 1 | 1 | 1 |
| Burkina Faso | 0 | 0 | 0.25 | 0.25 | | 0 | 0 | 0 |
| Cambodia | 0 | 0 | 0.5 | 1 | | 0 | 0 | 0 |
| Cameroon | #N/A | #N/A | #N/A | #N/A | | #N/A | #N/A | - |
| Canada | 1 | 1 | 1 | 1 | | 1 | 1 | 1 |
| Chile | 1 | 0 | 0.75 | 0.5 | | 0.75 | 0 | 1 |
| China | 0 | 0 | 0.75 | 0.75 | | 0 | 0 | 0 |
| Colombia | 1 | 1 | 0.75 | 0.75 | | 0.75 | 0.75 | 1 |
| Costa Rica | 1 | 1 | 0.25 | 0.75 | | 0.25 | 0.75 | 1 |
| Croatia | 1 | 1 | 1 | 1 | | 1 | 1 | 1 |
| Cyprus | 1 | 1 | 1 | 0.5 | | 1 | 0.5 | 1 |
| Czech Republic | 1 | 1 | 1 | 1 | | 1 | 1 | 1 |
| Denmark | 1 | 1 | 1 | 1 | | 1 | 1 | 1 |
| Ecuador | #N/A | #N/A | #N/A | #N/A | | #N/A | #N/A | - |
| Egypt | 0 | 0 | 0.75 | 0.5 | | 0 | 0 | 0 |
| El Salvador | #N/A | 1 | #N/A | 0.25 | | #N/A | 0.25 | - |
| Estonia | 1 | 1 | 1 | 1 | | 1 | 1 | 1 |
| Ethiopia | #N/A | #N/A | 0 | 0 | | #N/A | #N/A | - |
| Finland | 1 | 1 | 0.75 | 0.75 | | 0.75 | 0.75 | 1 |
| France | 1 | 1 | 1 | 1 | | 1 | 1 | 1 |
| Georgia | 0 | 0.75 | 1 | 1 | | 0 | 0.75 | 1 |
| Germany | 1 | 1 | 1 | 1 | | 1 | 1 | 1 |
| Ghana | #N/A | 0 | #N/A | #N/A | | #N/A | #N/A | - |
| Greece | 1 | 1 | 1 | 1 | | 1 | 1 | 1 |
| Guatemala | #N/A | #N/A | #N/A | #N/A | | #N/A | #N/A | - |
| Haiti | #N/A | #N/A | 0 | 0 | | #N/A | #N/A | - |
| Honduras | 0 | 0 | 0.75 | 0.75 | | 0 | 0 | 0 |
| Hungary | 1 | 1 | 1 | 0.25 | | 1 | 0.25 | 1 |
| Iceland | 1 | 1 | 1 | 1 | | 1 | 1 | 1 |
| India | 0 | 0 | 0.75 | 0.75 | | 0 | 0 | 0 |
| Indonesia | 0 | 0 | 1 | 1 | | 0 | 0 | 0 |
| Iran | 0.25 | 0 | 1 | 0.25 | | 0.25 | 0 | 1 |
| Iraq | 1 | 0.75 | 0.5 | 0.5 | | 0.5 | 0.375 | 1 |
| Ireland | 1 | 1 | 1 | 1 | | 1 | 1 | 1 |
| Israel | 1 | 1 | 1 | 1 | | 1 | 1 | 1 |
| Italy | 1 | 1 | 1 | 1 | | 1 | 1 | 1 |
| Jamaica | 0 | 0 | 0.75 | 0.75 | | 0 | 0 | 0 |
| Japan | 0.75 | 0.75 | 1 | 1 | | 0.75 | 0.75 | 1 |
| Kazakhstan | 1 | 1 | 1 | 0.25 | | 1 | 0.25 | 1 |
| Kenya | 0.25 | 0 | 0.25 | 0.75 | | 0.0625 | 0 | 0 |
| Kuwait | 1 | 1 | 1 | 1 | | 1 | 1 | 1 |
| Latvia | 1 | 0 | 1 | 1 | | 1 | 0 | 1 |
| Lebanon | 0.75 | 0.75 | 0.25 | 0.75 | | 0.1875 | 0.5625 | 1 |
| Liberia | #N/A | #N/A | 0 | 0 | | #N/A | #N/A | 0 |
| Libya | 0 | 0 | 0.5 | 0.25 | | 0 | 0 | 0 |
| Lithuania | 1 | 1 | 1 | 1 | | 1 | 1 | 1 |
| Luxembourg | 1 | 1 | 1 | 1 | | 1 | 1 | 1 |
| Madagascar | 0 | 0 | 0 | 0 | | 0 | 0 | 0 |
| Malawi | #N/A | #N/A | #N/A | #N/A | | #N/A | #N/A | 0 |
| Malaysia | 0 | 0 | 1 | 1 | | 0 | 0 | 0 |
| Malta | 0 | 0 | 0.75 | 0.75 | | 0 | 0 | 0 |
| Mexico | 0 | 1 | 0.75 | 0.25 | | 0 | 0.25 | 1 |
| Mongolia | #N/A | 0 | #N/A | 0.25 | | #N/A | 0 | 0 |
| Montenegro | 1 | 1 | 1 | 1 | | 1 | 1 | 1 |
| Morocco | 0 | 0 | 0.5 | 0.5 | | 0 | 0 | 0 |
| Myanmar | 0 | 0 | 1 | 1 | | 0 | 0 | 0 |
| Namibia | #N/A | #N/A | 0.25 | 0 | | #N/A | #N/A | 0 |
| Nepal | 0 | 0 | 0,75 | #N/A | | #VALUE! | #N/A | 0 |
| Netherlands | 1 | 1 | 1 | 1 | | 1 | 1 | 1 |
| New Zealand | 1 | 1 | 1 | 1 | | 1 | 1 | 1 |
| Nicaragua | 0 | 0 | 0.5 | 0.75 | | 0 | 0 | 0 |
| Niger | #N/A | #N/A | #N/A | #N/A | | #N/A | #N/A | 0 |
| Nigeria | #N/A | 0 | #N/A | 0.75 | | #N/A | 0 | 0 |
| Norway | 1 | 1 | 1 | 1 | | 1 | 1 | 1 |
| Oman | #N/A | #N/A | #N/A | #N/A | | #N/A | #N/A | 0 |
| Pakistan | 0 | 0.25 | 0.75 | 1 | | 0 | 0.25 | 1 |
| Papua New Guinea | #N/A | #N/A | 0 | 0 | | #N/A | #N/A | 0 |
| Peru | 0 | 0 | 0.75 | 0.75 | | 0 | 0 | 0 |
| Philippines | 0.25 | 0.25 | 1 | 1 | | 0.25 | 0.25 | 1 |
| Poland | 1 | 1 | 1 | 1 | | 1 | 1 | 1 |
| Portugal | 1 | 1 | 1 | 1 | | 1 | 1 | 1 |
| Qatar | 1 | 1 | 1 | 1 | | 1 | 1 | 1 |
| Republic of Korea | 0.75 | 0.75 | 1 | 1 | | 0.75 | 0.75 | 1 |
| Republic of Moldova | #N/A | #N/A | #N/A | #N/A | | #N/A | #N/A | 0 |
| Romania | 1 | 1 | 1 | 0.75 | | 1 | 0.75 | 1 |
| Russian Federation | 1 | 1 | 0.5 | 0.25 | | 0.5 | 0.25 | 1 |
| Rwanda | 0 | 0 | 0.25 | #N/A | | 0 | #N/A | 0 |
| Saudi Arabia | 1 | 1 | 1 | 1 | | 1 | 1 | 1 |
| Serbia | 1 | 0 | 1 | 0.5 | | 1 | 0 | 1 |
| Singapore | 0.5 | 0.5 | 1 | 1 | | 0.5 | 0.5 | 1 |
| Slovakia | 1 | 0 | 0.5 | 0.5 | | 0.5 | 0 | 1 |
| Slovenia | 1 | 1 | 1 | 1 | | 1 | 1 | 1 |
| Somalia | #N/A | #N/A | #N/A | #N/A | | #N/A | #N/A | 0 |
| South Africa | 0 | 0 | 0.5 | 0.5 | | 0 | 0 | 0 |
| Spain | 1 | 1 | 1 | 1 | | 1 | 1 | 1 |
| Sri Lanka | #N/A | #N/A | #N/A | #N/A | | #N/A | #N/A | 0 |
| Sudan | #N/A | #N/A | #N/A | #N/A | | #N/A | #N/A | 0 |
| Suriname | 0 | #N/A | 0.25 | #N/A | | 0 | #N/A | 0 |
| Sweden | 1 | 1 | 1 | 1 | | 1 | 1 | 1 |
| Switzerland | 1 | 1 | 1 | 1 | | 1 | 1 | 1 |
| Syria | #N/A | #N/A | #N/A | #N/A | | #N/A | #N/A | 0 |
| Thailand | 0 | 0 | 0.75 | 0.75 | | 0 | 0 | 0 |
| The Republic of North Macedonia | 1 | 1 | 0.75 | 0.75 | | 0.75 | 0.75 | 1 |
| Tunisia | 1 | 1 | #N/A | 0.75 | | #N/A | 0.75 | 0 |
| Türkiye | 0 | 0 | 1 | 1 | | 0 | 0 | 0 |
| Turkmenistan | 0 | 1 | 0 | 0.25 | | 0 | 0.25 | 1 |
| Uganda | 0 | 0 | 0 | 0 | | 0 | 0 | 0 |
| United Arab Emirates | 1 | 1 | 1 | 1 | | 1 | 1 | 1 |
| United Kingdom | 1 | 1 | 1 | 1 | | 1 | 1 | 1 |
| Tanzania | 0 | #N/A | 0.5 | #N/A | | 0 | #N/A | 0 |
| United States of America | 0.75 | 0.75 | 1 | 1 | | 0.75 | 0.75 | 1 |
| Uzbekistan | 0 | 0 | 0.75 | 0.75 | | 0 | 0 | 0 |
| Venezuela | 0 | 0 | 0.5 | 0.25 | | 0 | 0 | 0 |
| Viet Nam | 0 | 0 | 0.75 | 0.75 | | 0 | 0 | 0 |
| Yemen | 0.25 | 0 | 0.25 | 0.25 | | 0.0625 | 0 | 0 |
| Zambia | 0 | 0 | 0 | 0 | | 0 | 0 | 0 |
| Zimbabwe | #N/A | #N/A | #N/A | #N/A | | #N/A | #N/A | 0 |

Atezo=atezolizumab; Pembro=pembrolizumab

^†^The scores correspond to the categories on out-of-pocket costs reported by ESMO: “Free” = 1; “<25%” =0.75; “cost 25-50%” =0.5; “cost >50% but less than full cost” =0.25; “Full cost” =0; “Data not reported”=#N/A

^‡^ The scores correspond to the categories on accessibility reported by ESMO: “Always” =1; “Usually” =0.75; “Half the time” =0.5; “Occasionally” =0.25; “Never” =0; “Data not reported” = #N/A

^§^ Overall accessibility was the multiplication of the two scores.

^^^ Accessibility status is scored 1 if the combined score is ≥0.25.

**Estimating average person-months on treatment**

| The United States of America | | | | |
| --- | --- | --- | --- | --- |
| **Month** | **Nr people on treatment** | **Nr people discontinued** | **Average months on treatment for people who discontinued in the time period** | **Person-months on treatment** |
| 0 | 100 | 50 | 3.35 | 167.5 |
| 6.7 | 50 | 15.6 | 9.35 | 145.86 |
| 12 | 34.4 | 15.4 | 18 | 277.2 |
| 24 | 19 | 19 | 24 | 456 |
| 24 |  |  | Average person-months on treatment | 10.5 |

Source: Supplemental Figure 2, Velcheti et al^36^

| EU5: France, Germany, Italy, Spain, United Kingdom | | | | |
| --- | --- | --- | --- | --- |
| **Month** | **Nr people on treatment** | **Nr people discontinued** | **Average months on treatment for people who discontinued in the time period** | **Person-months on treatment** |
| 0 | 100 | 25 | 0.95 | 23.75 |
| 1.9 | 75 | 25 | 3.45 | 86.25 |
| 5 | 50 | 25 | 7 | 175 |
| 9 | 25 | 14.2 | 10.5 | 149.1 |
| 12 | 10.8 | 8.8 | 18 | 158.4 |
| 24 | 2 | 2 | 24 | 48 |
|  |  |  | Average person-months on treatment | 6.4 |

Source: Figure 2a, Slowley et al^36^

| Japan | | | | |
| --- | --- | --- | --- | --- |
| **Month** | **Nr people on treatment** | **Nr people discontinued** | **Average months on treatment for people who discontinued in the time period** | **Person-months on treatment** |
| 0 | 100 | 50 | 2.85 | 142.5 |
| 5.7 | 50 | 0.8 | 5.85 | 4.68 |
| 6 | 49.2 | 22.9 | 9 | 206.1 |
| 12 | 26.3 | 13.1 | 15 | 196.5 |
| 18 | 13.2 | 9.7 | 21 | 203.7 |
| 24 | 3.5 | 3.5 | 24 | 84 |
|  |  |  | Average person-months on treatment | 8.4 |

Source: Figure 2b, Goto et al^40^

**Mathematical representation of the main model components for expenditure projection**

Total projected expenditure is a function of volume (V) and cost (C) in country (*i*), and where applicable age groups (*j*) and year (*t*):

$$\sum_{i=1}^{194} V_{i,j,t}C_{i,j,t}$$

*Where*

$V_{i,j,t}$ *= population eligible for treatment × Access, testing and treatment coverage*

$C_{i,j,t}$*= price × vials × duration*

$V_{i,j,t}=\left[ X_{i,j,t}\cdot I_{i,j}\cdot{Pr}_{NSCLC}\cdot{PrAdv}_{i,j}\cdot(1-{PrEGFR}_{i,j})\cdot PrPDL \right]\cdot\left[ a_{i,j,t}\cdot{{PrTest}_{i}\cdot Tr}_{i,t} \right]$

*X*: Country population aged 40–74 years

*I* : Incidence rates of lung cancer

${Pr}_{NSCLC}$: Proportion of NSCLC among lung cancer cases

$PrAdv$: Proportion of advanced NSCLC unresectable at presentation

$PrEGFR$: Prevalence of EGFR mutation in NSCLC

$PrPDL$: Proportion PD-L1 TPS≥50% among EGFR-wildtype tumours

a: access to pembrolizumab; *a*∈{0,1}

Prtest: Proportion of patients receiving molecular tests

*Tr*: Treatment rate with immune checkpoint inhibitors

$C_{i,j,t}=P_{i,j,t}\cdot N_{i,t}\cdot D_{i,j}$

P: Ex-manufacturer price of pembrolizumab 100mg/4 mL vial

N: Number of vials of pembrolizumab

D: Average person-months on treatment

**Estimating PD1/PD-L1 Inhibitor expenditure as a proportion of total pharmaceutical spending**

The expenditure estimates for PD1/PD-L1 inhibitors (from the main model) were calculated as a percentage of total pharmaceutical expenditure derived from the reported total health expenditure and the share allocated to pharmaceuticals. Country-specific total health expenditure (in US dollars) was obtained from the WHO Global Health Expenditure Database (GHED).^65^ To match the modelling time frame of 2024–2040, total health expenditure was projected using a linear trend function in Microsoft Excel based on GHED data from 2018–2023. The model utilized reported proportions of health expenditure attributable to pharmaceuticals from multiple sources.^66–69^ In cases where country-specific data was unavailable, the model applied the average proportion based on the country's income level. Due to a lack of data for low-income countries, the model used the maximum proportion (49.3%) observed across all countries, based on the assumption that the share of pharmaceutical expenditure relative to total health expenditure tends to be higher in lower-income countries. Finally, the overall proportion for each country was calculated by summing the estimated expenditure on PD1/PD-L1 inhibitors and the total estimated pharmaceutical expenditure from 2024 to 2040.

**Model validation**

Technical validity and face validity were established by testing variables using extreme values (e.g. zero and 100%) and visualizing the results to assess model behaviour, allowing any unexpected model behaviour and formula errors to be identified and eliminated.

The predictive validity of the model was confirmed, as the estimated expenditure of US$2,634 million in 2025 based on the modelled population is within plausible range of independently derived estimates of US$2,454 million (range: 1,707 million–3,201 million). This independent estimate was based on the forecasted global sales of PD1/PD-L1 inhibitors in 2025 ($58 billion)^70^, 22.3% of those sales (in 2021) were attributed to NSCLC^70^, and the finding that about 23% (95% confidence interval: 0.16–0.30) of NSCLC patients had TPS≥50%^25,71^, with 82.5% of these patients having *EGFR-*wildtype status^25^. The global sales of PD1/PD-L1 inhibitors for NSCLC are expected to remain similar through 2028.^70^

**References**

1 United Nations Department of Economic and Social Affairs - Population Division. World population prospects 2022. Online Ed. 2022. https://population.un.org/wpp/.

2 Institute for Health Metrics and Evaluation. 2021 Global burden of disease study - GBD results tool. 2024. https://vizhub.healthdata.org/gbd-results/ (accessed Nov 15, 2024).

3 Zhang Y, Vaccarella S, Morgan E, *et al.* Global variations in lung cancer incidence by histological subtype in 2020: a population-based study. *Lancet Oncol* 2023; **24**: 1206–18.

4 Morgensztern D, Ng SH, Gao F, Govindan R. Trends in stage distribution for patients with non-small cell lung cancer: a National Cancer Database survey. *J Thorac Oncol* 2010; **5**: 29–33.

5 Casal-mouriño A, Ruano-ravina A, Lorenzo-gonzález M, *et al.* Epidemiology of stage III lung cancer: frequency, diagnostic characteristics, and survival. *Transl Lung Cancer Res* 2021; **10**: 506–18.

6 Nwagbara UI, Ginindza TG, Hlongwana KW. Health systems influence on the pathways of care for lung cancer in low- and middle- income countries : a scoping review. 2020; **8**: 1–11.

7 de Lima KYN, Cancela MDC, Leandro D. Spatial assessment of advanced-stage diagnosis and lung cancer mortality in Brazil. *PLoS One* 2022; **17**: 1–19.

8 Jazieh AR, Onal HC, Tan DS, Soo RA. Real-world global data on targeting epidermal growth factor receptor mutations in stage III non-small-cell lung cancer : the results of the KINDLE study. *Ther Adv Med Oncol* 2022; **14**: 1–17.

9 Arrieta O, Cardona AF, Martín C, *et al.* Updated frequency of EGFR and KRAS mutations in NonSmall-cell lung cancer in Latin America: The Latin-American consortium for the investigation of lung cancer (CLICaP). *J Thorac Oncol* 2015; **10**: 838–43.

10 Van Christ Manirakiza A, Rubagumya F, Rugengamanzi E, *et al.* Trends of molecular testing for lung cancer at the King Faisal Hospital, Kigali: Therapeutic and survival implications. *JTO Clin Res Reports* 2022; **3**: 100304.

11 Melosky B, Kambartel K, Häntschel M, *et al.* Worldwide prevalence of epidermal growth factor receptor mutations in non-small cell lung cancer: a meta-analysis. *Mol Diagnosis Ther* 2022; **26**: 7–18.

12 Leduc N, Ahomadegbe C, Agossou M. Incidence of lung adenocarcinoma biomarker in a Caribbean and African Caribbean population. *J Thorac Oncol* 2016; **11**: 769–73.

13 Rennert G, Gottfried M, Rennert HS, *et al.* Translational oncology long term follow-up of EGFR mutated NSCLC cases. *Transl Oncol* 2021; **14**: 10–5.

14 Boustany Y, Laraqui A, El Rhaffouli H, *et al.* Prevalence and patterns of EGFR mutations in non-small cell lung cancer in the Middle East and North Africa. *Cancer Control* 2022; **29**: 1–9.

15 Noronha V, Budukh A, Chaturvedi P, *et al.* Uniqueness of lung cancer in Southeast Asia. *Lancet Reg Heal - Southeast Asia* 2024; **27**: 100430.

16 Benbrahim Z, Antonia T, Mellas N. EGFR mutation frequency in Middle East and African non-small cell lung cancer patients: A systematic review and meta- analysis. *BMC Cancer* 2018; **18**: 891.

17 Martin C, Cuello M, Barajas O, *et al.* Real-world evaluation of molecular testing and treatment patterns for EGFR mutations in non-small cell lung cancer in Latin America. *Mol Clin Oncol* 2022; **16**: 1–10.

18 Aye PS, Mckeage MJ, Tin Tin S, Khwaounjoo P, Elwood M. Population-based incidence rates and increased risk of EGFR mutated non-small cell lung cancer in Māori and Pacifica in New Zealand. *PLoS One* 2021; **16**: e0251357.

19 Yessentayeva SY, Makarov VA, Kalmatayeva ZA, Zhakenova ZK, Arybzhanov DT. Molecular genetic tests in survival factors in patients with NSCLC in the clinical practice of Kazakhstan. *Med J Islam Repub Iran* 2021; **35**: 1–11.

20 Chan SW, Maske CP, Ruff P. EGFR mutations in non-small cell lung cancer in South Africa. *Ann Oncol* 2015; **26**: i1–i5.

21 Kumari N, Singh S, Haloi D, Kumar S. Epidermal growth factor receptor mutation frequency in squamous cell carcinoma and its diagnostic performance in cytological samples : a molecular and immunohistochemical study. *World J Oncol* 2019; **10**: 142–50.

22 Palacio S, Pontes L, Prado E, *et al.* EGFR mutation testing : changing patterns of molecular testing in Brazil. *Oncologist* 2019; **24**: e137–e141.

23 Yatabe Y, Frcpath KMK, Utomo A, *et al.* EGFR mutation testing practices within the Asia Pacific region: results of a multicenter diagnostic survey participating sites and study design. *J Thorac Oncol* 2015; **10**: 438–45.

24 Dietel M, Savelov N, Salanova R, *et al.* Lung Cancer Real-world prevalence of programmed death ligand 1 expression in locally advanced or metastatic non–small-cell lung cancer: The global , multicenter EXPRESS study. *Lung Cancer* 2019; **134**: 174–9.

25 Parra-medina R, Castañeda-gonzález JP, Montoya L, Gómez-gómez MP, Cabezas DC, Vargas MP. PD-L1 expression in non-small cell lung carcinoma in Latin America : a systematic review and meta-analysis. 2024; **13**: 1660–71.

26 Cherny N, ESMO collaborating partners. Insights in essential medicine availability and accessibility from ESMO antineoplastic medicines study 2.0. In: ESMO Congress. Madrid, 2023. https://www.esmo.org/newsroom/esmo-society-updates/universal-health-coverage-day-esmo-s-study-on-the-availability-of-anti-cancer-medicines-must-work-as-a-wake-up-call.

27 Smeltzer MP, Wynes MW, Lantuejoul S, *et al.* The International Association for the Study of Lung Cancer Global Survey on Molecular Testing in Lung Cancer. *J Thorac Oncol* 2020; **15**: 1434–48.

28 Kerr KM, Wolf J, Ohrling K, Burdon P, Malapelle U, Büttner R. Lung Cancer The evolving landscape of biomarker testing for non-small cell lung cancer in Europe. *Lung Cancer* 2021; **154**: 161–75.

29 Chambers P, Man KKC, Lui VWY, Mpima S, Nasuti P. Understanding molecular testing uptake across tumor types in eight countries : results from a multinational cross-sectional survey. *JCO Oncol Pract* 2020; **16**: e770–8.

30 How SH, Tho LM, Liam CK, *et al.* Programmed death-ligand 1 expression and use of immune checkpoint inhibitors among patients with advanced non-small-cell lung cancer in a resource-limited country. *Thorac Cancer* 2022; **13**: 1676–83.

31 Lee DH, Tsao M, Kambartel K, *et al.* Molecular testing and treatment patterns for patients with advanced non-small cell lung cancer : PIvOTAL observational study. *PLoS One* 2018; **27**: e0202865.

32 Carroll NM, Eisenstein J, Burnett-Hartman AN, *et al.* Uptake of novel systemic therapy: Real world patterns among adults with advanced non-small cell lung cancer. *Cancer Treat Res Commun* 2023; **36**: 100730.

33 Leung B, Shokoohi A, Al-Hashami Z, *et al.* Improved uptake and survival with systemic treatments for metastatic non-small cell lung cancer: younger versus older adults. *BMC Cancer* 2023; **23**: 1–11.

34 Noronha V, Abraham G, Patil V, *et al.* A real-world data of immune checkpoint inhibitors in solid tumors from India. *Cancer Med* 2021; **10**: 1525–34.

35 Griesinger F, Maurice P, Girard N, *et al.* Impact of immune checkpoint inhibitors on the management of locally advanced or metastatic non-small cell lung cancer in real-life practice in patients initiating treatment between 2015 and 2018 in France and Germany. *Lung Cancer* 2022; **172**: 65–74.

36 Velcheti V, Rai P, Kao YH, Chirovsky D, Nunes AT, Liu S V. 5-year real-world outcomes with frontline pembrolizumab monotherapy in PD-L1 expression ≥50% advanced NSCLC. *Clin Lung Cancer* 2024; **25**: 502-508.e3.

37 Slowley A, Phiri K, Multani JK, *et al.* Real-world treatment patterns and clinical outcomes after introduction of immune checkpoint inhibitors: Results from a retrospective chart review of patients with advanced/metastatic non-small cell lung cancer in the EU5. *Thorac Cancer* 2023; **14**: 2846–58.

38 Abraham G, Noronha V, Rajappa S, *et al.* The clinical utility and safety of short-course immune checkpoint inhibitors in multiple tumours — A real-world multicentric study from India. *Int J Cancer* 2022; **150**: 1045–52.

39 Goto Y, Kawamura K, Fukuhara T, *et al.* Health care resource use among patients with advanced non–small cell lung cancer in Japan, 2017–2019. *Curr Ther Res - Clin Exp* 2023; **99**: 100712.

40 Goto Y, Tamura A, Matsumoto H, Taniguchi K, Kamitani T, Irisawa M. First-line pembrolizumab monotherapy for advanced NSCLC with programmed death-ligand 1 expression greater than or equal to 50 %: Real-world study including older patients in Japan. *JTO Clin Res Reports* 2022; **3**: 100397.

41 Merck & Co. Inc. KEYTRUDA® (pembrolizumab) injection, for intravenous use. New Jersey: Merck, 2024.

42 Malmberg R, Zietse M, Dumoulin DW, *et al.* Alternative dosing strategies for immune checkpoint inhibitors to improve cost-effectiveness: a special focus on nivolumab and pembrolizumab. *Lancet Oncol* 2022; **23**: e552–61.

43 Low JL, Huang Y, Sooi K, *et al.* Low-dose pembrolizumab in the treatment of advanced non-small cell lung cancer. *Int J Cancer* 2021; **149**: 169–76.

44 NHS England. National Dose Banding Table – Pembrolizumab 25 mg/mL. 2022. https://www.england.nhs.uk/publication/national-dose-banding-table-pembrolizumab-25-mgml/ (accessed Nov 15, 2024).

45 Canadian Health Technologies Agency. CADTH reimbursement recommendation- Pembrolizumab (Keytruda). *Canada J Heal Technol* 2022; **2**.

46 Bonomi P, Moudgalya H, Gomez SL, *et al.* Frequency of weight and body composition increases in advanced non-small cell lung cancer patients during first line therapy. *J Cachexia Sarcopenia Muscle* 2024; **15**: 2311–22.

47 NCD Risk Factor Collaboration (NCD-RisC). Worldwide trends in underweight and obesity from 1990 to 2022: a pooled analysis of 3663 population- representative studies with 222 million children, adolescents, and adults. *Lancet* 2024; **403**: 1027–50.

48 Australian Government Deparment of Health and Aged Care. Pembrolizumab. Pharm. Benefits Scheme. 2024. https://www.pbs.gov.au/browse/downloads (accessed Dec 11, 2024).

49 Institut National d’Assurance Maladie Invalidité. Pembrolizumab. Médicaments Prod. radio-pharmaceutiques Rembours. 2024. https://webappsa.riziv-inami.fgov.be/ssp/ProductSearch (accessed Dec 11, 2024).

50 Câmara de Regulação do Mercado de Medicamentos. Pembrolizumab. List. PREÇOS Medicam. - PREÇOS FÁBRICA E MÁXIMOS VENDA AO Gov. 2024.

51 National Council on Prices and reimbursement of medicinal products R of B. Pembrolizumab. Posit. drug List. Annex 4. 2024.

52 Gobierno de Chile. Pembrolizumab. Chile Cent. Abastecimiento del Sist. Nac. Serv. Salud. 2024. https://www.cenabast.cl/compras-cenabast/ (accessed Dec 11, 2024).

53 National Pharmaceutical Pricing Authority of India. Pembrolizumab. PHARMA SAHI DAAM. 2024. https://nppaipdms.gov.in/NPPA/PharmaSahiDaam/searchMedicine (accessed Dec 13, 2024).

54 Japan Ministry of Health, Labour, and Welfare . 注射薬 [Injectables]. 薬価基準収載品目リスト及び後発医薬品に関する情報について（令和6年12月6日適用） [Information drug price List generic drugs (effective December 6, 2024)]. 2024. https://www.mhlw.go.jp/topics/2024/04/tp20240401-01.html (accessed Dec 11, 2024).

55 Greece Ministry of Health. Pembrolizumab. Updat. Drug Price List. Inc. Adm. Chang. 2024. https://www.moh.gov.gr/articles/times-farmakwn/deltia-timwn.

56 National Department of Health of South Africa. Database of Medicine Prices 11 July 2022. South African Med. Price Regist. 2024. http://www.mpr.gov.za/PublishedDocuments.aspx#DocCatId=21 (accessed Dec 11, 2024).

57 Republic of Türkiye Ministry of Health. KEYTRUDA 100 mg/4ml (25 mg/ml) Infuzyonluk Cozelti. Ref. Bazlı İlaç Fiyat List. 2024. https://titck.gov.tr/dinamikmodul/100 (accessed Dec 13, 2024).

58 Sheffield BS, Eaton K, Emond B, *et al.* Cost savings of expedited care with upfront next-generation sequencing testing versus single-gene testing among patients with metastatic non-small cell lung cancer based on current Canadian practices. *Curr Oncol* 2023; **30**: 2348–65.

59 Australian Government Department of Health and Aged Care. Amendment to pathology MBS item 72814 for programmed cell death ligand ( PD-L1 ) testing. Medicare Benefits Sched. 2022; published online Sept 20.

60 Roy A. PD-L1 Testing in India. What is PL-L1, Where to get tested, and Cost of PD-L1 Test. 4baseCare. 2024. https://4basecare.com/pd-l1-testing-in-india/ (accessed Dec 6, 2024).

61 Loong HH, Wong CKH, Leung LKS, *et al.* Cost effectiveness of PD-L1-based test-and-treat strategy with pembrolizumab as the first-line treatment for metastatic NSCLC in Hong Kong. *PharmacoEconomics - Open* 2020; **4**: 235–47.

62 Cheng S, Pei R, Li J, *et al.* Atezolizumab compared to chemotherapy for first-line treatment in non-small cell lung cancer with high PD-L1 expression : a cost-effectiveness analysis from US and Chinese perspectives. 2021; **9**. DOI:10.21037/atm-21-4294.

63 Mino-kenudson BM, Lantuejoul S. Global Survey for Pathologists on PD-L1 Testing: Moving Toward Standardization. IASLC Lung Cancer News. 2019; : 61–2.

64 Cherny NI, Trapani D, Galotti M, *et al.* ESMO Global Consortium Study on the availability, out-of-pocket costs , and accessibility of cancer medicines: 2023 update. *Ann Oncol* 2024; **In press**. DOI:10.1016/j.annonc.2024.12.005.

65 World Health Organization. Current Health Expenditure (CHE), in million current US$. WHO Glob. Heal. Expend. Database. 2024. https://apps.who.int/nha/database (accessed Jan 15, 2024).

66 Organization for Economic Cooperation and Development. % of health spending. Pharm. Spend. 2021. https://www.oecd.org/en/data/indicators/pharmaceutical-spending.html (accessed Jan 15, 2025).

67 World Health Organization Regional Office for the Western Pacific. How pharmaceutical systems are organized in Asia and the Pacific. Manila, 2018 https://www.who.int/publications/i/item/9789290618485.

68 Kanavos P, Tzouma V, Fontrier A, Kamphuis B, Parkin GC, Saleh S. Pharmaceutical pricing and reimbursement in the Middle East and North Africa region A mapping of the current landscape and options for the future. London: LSE Consulting, 2018.

69 Dahmani H, Fradi I, Achour L, Toumi M. Pharmaceutical pricing and reimbursement policies in Algeria , Morocco , and Tunisia : comparative analysis. *J Mark Access Heal Policy* 2023; **11**. DOI:10.1080/20016689.2023.2244304.

70 Gores M. In the eye of the storm: PD-(L)1 inhibitors weathering turbulence - Challenges and opportunities as the immune checkpoint inhibitor market matures. Durham, North Carolina, 2022.

71 Haslam A, Prasad V. Estimation of the percentage of US patients with cancer who are eligible for and respond to checkpoint inhibitor immunotherapy drugs. *JAMA Netw Open* 2019; **2**: 1–9.
